# Understanding the autophagic functions in cancer stem cell maintenance and therapy resistance

**DOI:** 10.1017/erm.2024.23

**Published:** 2024-10-08

**Authors:** Niharika, Minal Garg

**Affiliations:** Department of Biochemistry, University of Lucknow, Lucknow 226007, India

**Keywords:** autophagy, cancer stemness, therapeutic resistance, tumour microenvironment, tumoural heterogeneity

## Abstract

Complex tumour ecosystem comprising tumour cells and its associated tumour microenvironment (TME) constantly influence the tumoural behaviour and ultimately impact therapy failure, disease progression, recurrence and poor overall survival of patients. Crosstalk between tumour cells and TME amplifies the complexity by creating metabolic changes such as hypoxic environment and nutrient fluctuations. These changes in TME initiate stem cell-like programmes in cancer cells, contribute to tumoural heterogeneity and increase tumour robustness. Recent studies demonstrate the multifaceted role of autophagy in promoting fibroblast production, stemness, cancer cell survival during longer periods of dormancy, eventual growth of metastatic disease and disease resistance. Recent ongoing studies examine autophagy/mitophagy as a powerful survival strategy in response to environmental stress including nutrient deprivation, hypoxia and environmental stress in TME. It prevents irreversible senescence, promotes dormant stem-like state, induces epithelial–mesenchymal transition and increases migratory and invasive potential of tumour cells. The present review discusses various theories and mechanisms behind the autophagy-dependent induction of cancer stem cell (CSC) phenotype. Given the role of autophagic functions in CSC aggressiveness and therapeutic resistance, various mechanisms and studies based on suppressing cellular plasticity by blocking autophagy as a powerful therapeutic strategy to kill tumour cells are discussed.

## Introduction

Despite treatment advancements, cancer continues to be a leading cause of high mortality rates of the patients who are diagnosed with advanced tumours. Surgical removal of tumours is the preferred choice of treatment, nevertheless it often fosters aggressive tumour relapse in case of metastatic tumours. Chemo- and/or radiotherapies impose multiple side effects and offer only transient eradication of tumours. Tumour recurrence and drug resistance are explored as the main reasons of therapy failure. They make the conventional therapies not only ineffective in targeting advanced tumours but also promote tumour regrowth. Recent studies examine the intratumoural heterogeneity being majorly responsible for therapy failure, disease progression, recurrence and poor overall survival of patients (Ref. 1).

**Figure 1.**
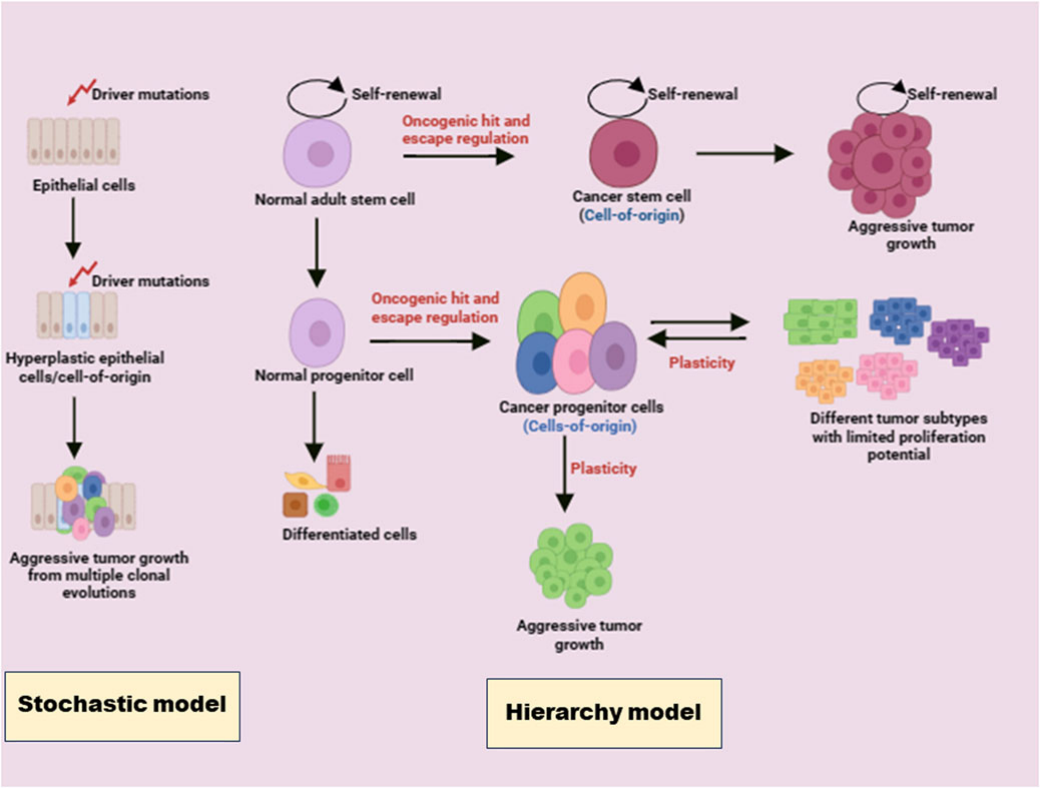
Models of tumourigenesis: (a) stochastic model – unique driver mutations produce tumour cells. Every tumour cell with an equal ability to act as cell-of-origin contributes to the genetically different subclone and thus brings about tumoural heterogeneity. (b) Hierarchy model – oncogenic hit turns normal adult stem cells and normal progenitor cells into cancer stem cells (CSCs) and cancer progenitor cells respectively. A small population of stem cells called CSCs contribute to aggressive tumour growth. Epithelial–mesenchymal plasticity aggravates tumour growth.

Two models of tumourigenesis stochastic model and hierarchy model help us to understand the concept of tumour progression and tumour heterogeneity. According to stochastic model, every cell within a tumour has an equal potential to be of cell-of-origin and facilitates tumour initiation and progression. Unique driver mutations result in the formation of genetically distinct subclones through branching evolution, thereby contribute to functional heterogeneity and impact the cancer hallmarks differently (Ref. 2). Besides genetic factors, there are strong emerging evidences regarding the contributory role of non-genetic determinants on tumoural heterogeneity (Fig. 1). These are largely related to developmental pathways and epigenetic modifications (DNA methylation, chromatin openness, histone modification, microRNA (miR), and other non-coding RNA) (Refs 3, 4).

According to hierarchy model, tumour progression occurs when long-lived adult stem cells generate cellular progeny throughout their life and produce multiple specialized, short-lived cells that can perform tissue-specific functions (Fig. 1). They escape regulation and give rise to stem cell-like counterpart called cancer stem cells (CSCs). This side population of cells, also called CSCs, constitutes less than 1% of cellular population. These cells predominantly reside within hypoxic, low pH and less nutrient niches. CSCs are known for self-renewal property, multipotency, potential to grow as spheres under serum deprivation and high aldehyde dehydrogenase (ALDH) activity, evasion of cell killing in part because of their quiescent state, increased expression of drug transporters and other resistance genes and intense tumourigenic potential (Refs 5, 6). CSCs derive energy from metabolic pathways for maintaining self-renewal, differentiation and tumourigenic potential (Ref. 7). CSCs maintain homoeostasis by relying predominantly on glycolysis (Ref. 8). However, few studies observe that many CSCs are more inclined towards oxidative phosphorylation (OXPHOS) than glycolysis for energy requirements. Higher oxidative potential and adenosine triphosphate (ATP) levels are observed with glioblastoma stem cells (GSCs) compared with differentiated glioma cells (Ref. 9). Similarly, breast CSCs exhibit reduced lactate production and increased ATP levels (Ref. 10). Tumour microenvironment (TME) thus provides a favourable metabolic environment to support the growth of CSCs.

**Figure 2.**
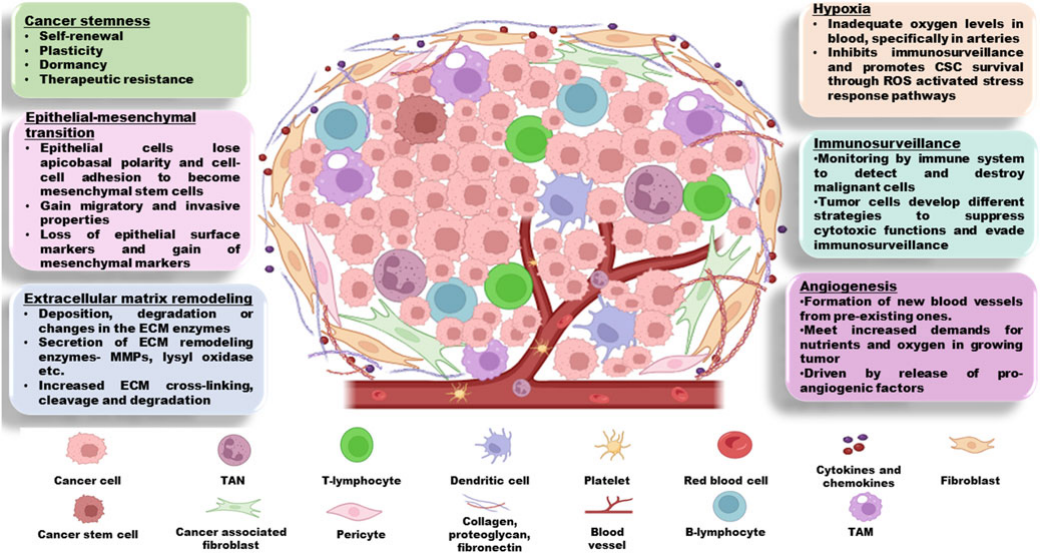
TME – a complex extracellular hypoxic environment comprises infiltrating endothelial, haematopoietic and perivascular cells, immune cells (TAM, TAN, lymphocytes and dendritic cells), CAFs, cytokines, growth factors and ECM components. This complex regulatory network supports tumour growth, angiogenesis, EMT and ECM remodelling. CAFs, cancer-associated fibroblasts; ECM, extracellular matrix; EMT, epithelial–mesenchymal transition; TAM, tumour-associated macrophages; TAN, tumour associated neutrophil; TME, tumour microenvironment.

Complex tumour ecosystem which comprises tumour cells and its associated TME constantly influences the tumoural behaviour and ultimately impacts the therapy failure (Ref. 11). TME consists of infiltrating endothelial, haematopoietic and perivascular cells or their progenitors, cancer-associated fibroblasts (CAFs), immune cells, extracellular matrix (ECM) components and stroma containing networks of cytokines and growth factors (Ref. 12) (Fig. 2). Crosstalk between tumour cells and TME amplifies the complexity by creating metabolic changes such as a hypoxic environment and nutrient fluctuations. These changes not only contribute to tumoural heterogeneity and increase the tumour robustness but also make the tumour cells resistant to drug responses (Refs 4, 5). Recent studies demonstrate the role of TME to initiate stem cell-like programmes in cancer cells. Depending upon the genotype and interaction with microenvironmental signals, transit-amplifying/progenitor cells undergo dedifferentiation and enter back into CSC pool and regain long-term tumour repopulation capacity. Tumour heterogeneity, relapse of therapy-resistant disease and metastatic dissemination in many different human cancers are attributed to the properties of CSCs (Ref. 13). Enriching our current understanding about the mechanisms responsible for cancer stemness and related progression of disease relapse crisis is the need of an hour for overcoming the therapy resistance.

Recent studies have examined the multifaceted role of autophagy in cancer cell survival during longer periods of dormancy and the eventual growth of metastatic disease (Ref. 14). Autophagy plays a central role in TME where it is induced in CAFs by their association with tumour cells, supplies recycled metabolites and promotes fibroblast production. Further, over the past few years, autophagy is shown to promote stemness, CD44 expression, targeted degradation of key transcription factors, such as p53 and forkhead boxO3A (FOXO3A), induces pluripotency, dormancy and drug resistance (Refs 15, 16, 17, 18). Elevated expression of autophagic markers such as ATG5 and Beclin1, an indicator of increased autophagic flux is observed in CSCs. Application of autophagic inhibitors significantly result in a decrease in autophagic flux and corresponding reduction in the number of CSCs (Ref. 19). Another study describes the detrimental effects of impaired mitophagy on the survival of CSCs (Ref. 20). These observations suggest the important role of autophagy in regulating the multifarious functions of CSCs. This review provides a recent update on how the TME promotes autophagy which further contributes to increased cancer stemness, dormancy and drug resistance. The present review further provides an insight into its clinical relevance with an aim to explore the possible therapeutic benefits including complete eradication of residual tumour cells and prevention of tumour relapse.

## Cancer stem cells

Tumours are composed of hierarchy of cell types where tumour-initiating cells (TICs) or CSCs are highly tumourigenic and are the source of tumour initiation and heterogeneity. They give rise to intermediate progenitors and terminally differentiated progeny. CSCs were first demonstrated in 1997 in acute myeloid leukaemia (AML) patients by transplantation of AML-initiating cell population into severe combined immune-deficient (SCID) mice as subset of cells. Leukaemia-initiating cells were enriched on the basis of expression of cell surface markers (CD34^+^/CD38^−^). These cells harbour the potential of self-renewal, propagation and differentiation (Ref. 21). Few of the characteristic features of CSCs are increased expressions of cluster of differentiation 44 (CD44^+^), CD133^+^, ATP-binding cassette (ABC) transporters, epithelial cell adhesion molecule and aldehyde dehydrogenase 1 (ALDH1). Since then, their existence has been shown in many cancers including breast, prostate, lung, brain, haematopoietic, head and neck, colon, skin and pancreatic cancers as well as in sarcomas. Notably, as few as 100 CSCs are identified in non-obese diabetic/SCID mice (Ref. 22). Transcriptional signatures and heterogeneously expressed cell surface markers specific to CSCs not only allow the accurate flow cytometric sorting of marker-positive and -negative subsets in a tumour population but also correlate with their aggressive behaviour and are highly predicative of overall patient survival (Table 1) (Refs 23, 24, 25, 26, 27, 28, 29, 30, 31, 32, 33, 34, 35, 36, 37, 38, 39, 40, 41, 42, 43, 44, 45, 46, 47, 48, 49, 50, 51, 52, 53, 54, 55, 56, 57, 58, 59, 60, 61, 62, 63).

**Table 1.** Tumourigenic properties of cancer stem cell markers in various cancer types

Tumour type	Marker	Tumourigenic functions	References
Colorectal, bladder, breast, liver, glioblastoma, pancreatic, head and neck, lung	CD44^+^	Cell survival, proliferation, cytoskeletal changes, cellular motility, EMT regulation	23, 24, 25, 26, 27, 28, 29, 30, 31, 32, 33, 34
Lung, liver, pancreatic	CD24^+^	Proliferation, adhesion and metastasis	28, 35, 36
Liver, pancreatic, colorectal, glioblastoma, head and neck, prostate and lung	CD133^+^	Self-renewal, tumourigenicity, therapeutic resistance, invasiveness	23, 24, 28, 29, 33, 34, 35, 36, 37, 38
Pancreatic, colorectal, liver, prostate	EpCAM	Self-renewal, invasiveness, tumourigenicity, therapeutic resistance	23, 28, 29, 33, 34, 39
Liver, pancreatic, lung, colorectal	ATP-binding cassette transporters	Therapeutic resistance by efflux of therapeutic agents	23, 29, 38, 40
Liver, breast, colorectal, bladder, cervical, head and neck, lung, prostate, leukaemia	ALDH1	Drug metabolism leading to therapeutic resistance, tumourigenicity	23, 25, 27, 28, 31, 33, 36, 41, 42
Liver, prostate, lung, head and neck	Bcl2	Invasion, metastasis	43, 44
Glioblastoma, liver, prostate	Vimentin	Mesenchymal-marker, invasion	29, 31, 45, 46, 47
Liver, colorectal, cervical	NANOG	Tumourigenesis	32, 36, 43, 48, 49
Glioblastoma, bladder, pancreatic	HIF1-*α*/HIF2-*α*	Cell metabolism, therapeutic resistance	37, 45, 46, 48, 49
Liver, breast, pancreatic, colorectal, bladder, cervical, prostate, head and neck, lung, leukaemia	Snail	EMT	51
Breast, cervical, colorectal	Oct4	Tumourigenesis, therapeutic resistance	23, 32, 36, 48, 52
Breast, leukaemia, pancreatic, prostate, bladder	NF-*κ*B	Cancer initiation, metastasis, therapeutic resistance	53, 54, 55, 56, 57
Triple negative breast cancer, lung, ovarian, gastric, colorectal, oesophageal	CXCR2	Proliferation, metastasis, therapeutic resistance	58, 59, 60
Liver, meningioma, gastric	CD13^+^	Angiogenesis, invasion, metastasis	61, 62, 63

ALDH1, aldehyde dehydrogenase 1; Bcl2, B-cell lymphoma 2; CD13, cluster of differentiation 13; CD24, cluster of differentiation 24; CD44, cluster of differentiation 44; CD133, cluster of differentiation 133; CXCR2, C–X–C motif chemokine receptor 2; EpCAM, epithelial cell adhesion molecule; EMT, epithelial–mesenchymal transition; HIF1-*α*/HIF2-*α*, hypoxia inducible factor 1 subunit *α*/hypoxia inducible factor 2 subunit *α*; NF-*κ*B, nuclear factor-kappa B; Oct4, octamer-binding transcription factor 4.

One of the theories regarding the origin of CSCs believes that normal stem cells/progenitor cells give rise to CSCs when encounter a specific genetic mutation or altered environment. The other theory believes that genetic or heterotypic alterations in somatic cells turn them into cancer cells with stem-like characteristics. Differences in the driver mutations and cell-of-origin create diversity in the CSC model in different cancer types and influence cancer properties (Ref. 64). A study on ependymomas explains the impact of different mutations on differences in gene expression and prognosis (Ref. 65). Changes in mutation spectrum with age in human leukaemias influence the frequency and phenotype of leukaemia-initiating cells (Ref. 66). Phenotypically diverse TICs are identified in solid tumours also. Breast cancer-initiating cells with surface markers CD44^+^CD24^−^/low does not universally distinguish tumourigenic and non-tumourigenic breast cancer cells (Ref. 22). Tumourigenic cells with different surface marker phenotypes because of difference in transforming mutations are examined in mouse models of lung cancer (Ref. 67).

Among the genomic alterations, epithelial–mesenchymal transition (EMT) induction, an epigenetic process is currently being explored as the mechanism of CSC generation. It places epithelial cells into quasi-mesenchymal states and allows the cancer cells to gain stem-like characteristics. EMT induction in immortalized human mammary epithelial cells is shown to increase their ability to form mammospheres (Ref. 68). Process of EMT is orchestrated by EMT-activating transcription factors including zinc-finger E-box-binding homoeobox (ZEB1), Smad (ZEB2), Snail1 (Snail), Snail2 (Slug) and Snail3 (Smuc), Twist1 and Twist2 and E12/E47 and Tbx3. Altered activities of Wnt, transforming growth factor-*β* (TGF-*β*), hedgehog, Notch, phosphatidylinositol 3 kinase/protein kinase B/mammalian target of rapamycin (PI3K/Akt/mTOR), signal transducer and activation of transcription 3 (STAT3) and nuclear factor-kappa B signalling pathways induce EMT. Activation of intracellular signalling pathways and the transcription factors including octamer-binding transcription factor 4 (OCT4), Sry-related HMG box 2 (SOX2), Kruppel-like factor 4 (KLF4), NANOG and cellular-myelocytomatosis (c-MYC) regulate the cancer stemness and promote tumourigenicity and cell survival in response to cancer treatments (Ref. 69).

There are studies which suggest that cells which acquire stemness character during EMT induction are lost in the course of complete EMT. However, cells maintain stem-like phenotype during partial EMT and exhibit plasticity. Spectra of E/M states were examined in xenografts obtained from breast, lung, oesophagus small cell carcinoma patients (Ref. 70). Subset of cancer cells with partial E/M or hybrid E/M phenotype co-express E-cadherin and vimentin and are observed to exhibit stem-like characteristics, higher tumourigenic potential and worse prognosis in skin squamous cell carcinoma, mammary tumours and triple negative breast cancer (Refs 69, 70, 71).

Studies establish that EMT programmes regulate a dynamic switch between CSC state (dedifferentiated/retrodifferentiated (dormant)) and non-CSC state (differentiated or proliferated). This reversible transition between CSC and non-CSC states identified as CSC plasticity is associated with worst disease-free survival and short overall survival of breast cancer patients (Ref. 72). Non-CSC state is characterized with fast cycling and drug susceptible phenotype whereas CSC state is identified with slow-cycling/dormant and drug refractory phenotype. Cycling CSCs possess epithelial phenotype, have replicative potential, express cytokine receptors and produce cytokines. On the other hand, non-cycling CSCs possess mesenchymal phenotype and have invasive/metastatic potential (Refs 58, 73). Reversible transition of phenotype could possibly result in drug-proactive behavioural changes in tumour cells and augment chemotherapy.

### Role of TME in promoting cancer stemness

Expansion of neoplastic cells comprising CSCs within TME creates tumour niche. CSCs constantly interact with the components of TME (CAFs, tumour vasculature, immune cells, other differentiated cells and extracellular cues) to remodel TME and maintain niche. Growth factors, cytokines and small RNAs in the local tissue environment are important for cell nutrition, signalling transduction, intercellular communication and cell fate and regulate CSC self-renewal, differentiation, tumourigenesis and metastasis. Stresses in TME-like hypoxia and TGF-*β* promote EMT, upregulate cell surface markers, increased self-renewal gene expression programmes and tumour-propagating properties (Ref. 74).

Mesenchymal stromal cells (MSCs) present in the TME of solid tumour mass are also known as CAFs. Tissue remodelling through ECM deposition, expression of matrix-associated proteolytic enzymes and dysregulated angiogenesis potentially increase the number of MSCs (Ref. 75). These cells secrete growth factors that bind to the surface of tumour cells and pro-angiogenic factors (vascular endothelial growth factor (VEGF) and platelet-derived growth factor) and promote tumour niche neovascularization and tumour growth. MSCs are involved in immunosuppressive functions. Higher expression of CD73 on the surface of MSCs favours the hydrolysis of adenosine monophosphate (AMP). Increased amounts of adenosine in the TME activate the immunosuppressive A2A adenosine receptor on CD8^+^ anti-tumour T cells and natural killer (NK) cells and dampen the immune response (Ref. 76). MSCs are also known to facilitate EMT by secreting chemokines and TGF-*β*. TGF-*β* is shown to control the self-renewal and differentiation properties of glioma-initiating cells derived from glioblastoma multiforme patients (Ref. 77). Recent studies examine the reciprocal crosstalk between CSCs and CAFs in the TME. Secretion of cytokines and growth factors (chemotactic factor-like C–C motif ligand 2 (CCL2), hepatocyte growth factor) induces various stemness regulators (such as Wnt and NOTCH), reprogrammes normal fibroblasts into CAFs and thus contributes to cancer stemness (Ref. 78). Medulloblastomas either arise from the activation of the sonic hedgehog pathway in granule neuron precursors of the cerebellum or from activation of the Wnt pathway in the dorsal brainstem progenitors (Refs 79, 80). Other than medulloblastoma, many cancers such as lymphoma, leukaemia, breast, gastric and colorectal cancer are studied for the role of mutations in the mediators of the Wnt pathway in the maintenance of CSC phenotype (Ref. 81). Hedgehog signalling is examined to play an important role in maintaining CSC phenotype in various cancer types including basal cell carcinoma, multiple myeloma, chronic myeloid leukaemia (CML), glioblastoma and colon cancer (Ref. 82). Role of NOTCH signalling in the regulation of tumour-initiating cells is demonstrated in cancers including leukaemia, glioblastoma, breast, colon, pancreas and lung (Ref. 83).

Release of angiogenic factors and their binding to the surface of endothelial cells (ECs) of nearby blood vessels in the prevailing acidic and hypoxic conditions in tumour niche initiates tumour angiogenesis. Further cytokines, growth factors, ECM proteins and ECM remodelling enzymes regulate the vascular ECs and promote the growth of abnormal tumour vasculature. Abnormal vasculature includes excessive branching, abnormal bulges, discontinuous EC lining and defective basement membrane (Ref. 84). Reciprocal interaction of CSCs with perivascular niche including ECs and ECM components is well documented. ECs promote the proliferation and self-renewal potential of CSCs via activation of signalling pathways including sonic hedgehog, NOTCH, nitric oxide, Jagged-1 and VEGF, neuropilin 1 and the secretion of pro-angiogenic factors (Refs 85, 86, 87, 88, 89, 90). On the other hand, CSCs stimulate endogenous ECs and drive tumour vascularization in many solid tumours including colorectal cancer, breast cancer, glioma and melanoma (Ref. 90).

Infiltration of immune cells in the TME of solid tumours is reported to have tumourigenic effects. Among the tumour infiltrating immune cells, tumour-associated macrophages (TAMs) form a dominant population whereas T-cells constitute a lower fraction in many tumour types. TAMs derived from circulating monocytes are recruited and reprogrammed in TME in response to chemokines, pro-inflammatory signals and damage-associated molecule patterns (DAMPs) with high mobility group box 1 (HMGB1) (Refs 91, 92). Binding of DAMPs to their specific pattern-recognition receptors on macrophages such as Toll-like receptor 4 (TLR4) with M1 phenotype triggers pro-inflammatory signals and anti-tumourigenic response. Nevertheless, upon arrival in TME, monocytes differentiate, polarized to an alternatively activated state and gain M2 phenotype. Such TAMs secrete chemokines and ligands, promote EMT and maintain cell stemness in many cancer types (Ref. 90).

Niche within the TME is critical for the maintenance of principal properties of CSCs, preserving their phenotype, providing protection from immunosurveillance and facilitating their metastatic potential. Recent studies examine the central role of autophagy in the niche of TME where CSCs reside. Autophagy is examined as an evolutionary conserved adaptive catabolic process which supports the viability of cells under environmental stress stimuli and maintains energy homoeostasis. CSCs are reported to be in autophagic state where autophagy helps them to survive during metastatic spreading. Given the multifaceted role of autophagy in cancer and its functional link with cancer stemness, it may be important to examine the functional role of autophagy in CSCs, maintenance of tumour cell dormancy and the mechanisms of cancer drug resistance.

## Autophagy and maintenance of cancer stemness

**Figure 3.**
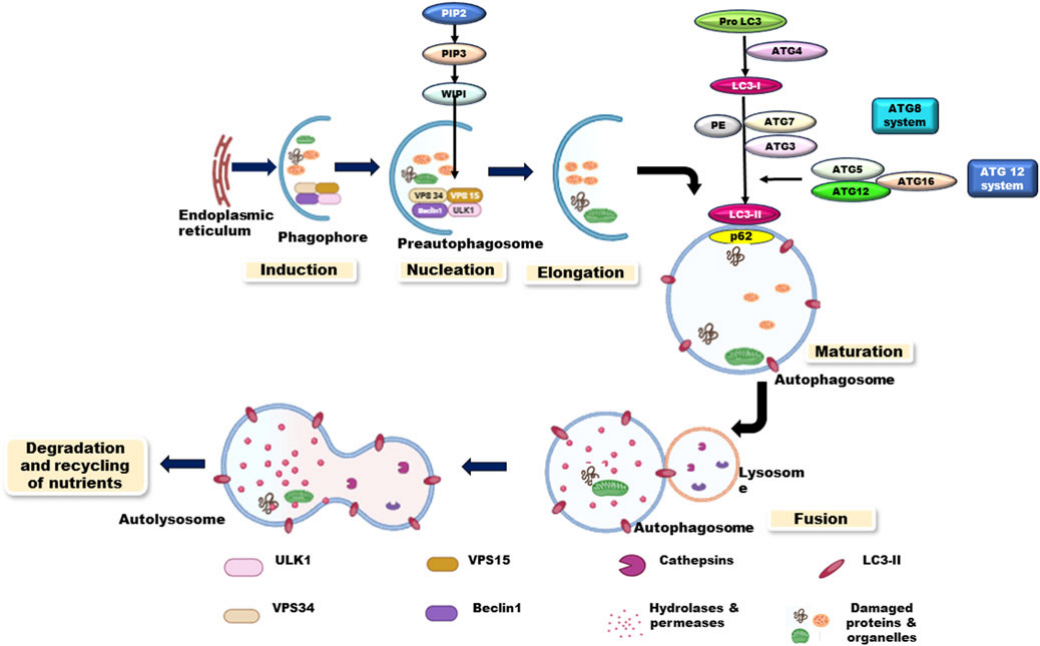
Process of macroautophagy.

Cells adapt autophagy as a powerful survival strategy in response to environmental stress including nutrient deprivation, hypoxia and environmental stress. Autophagy is a highly conserved catabolic process and it takes place in all eukaryotic cells. It is the process of lysosomal degradation wherein damaged, cytoplasmic proteins and organelles are eaten up by double-membrane autophagic vesicles known as autophagosomes (Ref. 93). Fusion of an autophagosome with a lysosome results in the formation of an autolysosomes. Acidic environment is maintained because of the presence of hydrolytic enzymes in autolysosomes which degrade the internalized cell additives (Ref. 94) (Fig. 3). Selective degradation of mitochondria, ribosomes and pathogens via autophagic process is also termed mitophagy, ribophagy and xenophagy respectively.

Three subtypes of autophagy in mammals have been identified: macroautophagy, microautophagy and chaperone-mediated autophagy (CMA). Macroautophagy is the principal autophagic pathway wherein the cell develops a double-membrane structure termed phagophore, which later develops into an autophagosome. Subsequently, cytoplasmic additives are engulfed into autophagosomes and brought to the lysosomes for binding and degradation. Microautophagy is characterized by the autonomous modification of the membrane shape of lysosome through invagination to trap cytoplasmic content directly. Number of suggested functions of microautophagy includes the maintenance of organellar size and composition of biological membrane. It regulates the composition of lysosomal/vacuolar membrane by allowing the incorporation of degraded lipids into vesicles. It allows the delivery of glycogen into lysosomes, maintains membrane proteins turnover and allows the cells to survive under nitrogen-restricted conditions. Microautophagy initiates with the membrane invagination and formation of autophagic tubes mediated by Atg7-dependent ubiquitin-like conjugation or via vacuolar transporter chaperone molecular complex. Lipid-enriched autophagic tubes/membranes promote vesicle formation and this is followed by vesicle enlargement mediated by binding enzymes in unclosed vesicles. Vesicles freely lying in the lumen undergo degradation by the activity of hydrolases and the nutrients are then released (Ref. 95).

Selective elimination of mitochondria by the process of mitophagy is primarily known in yeast and is induced by atg32. Toxic by-products such as reactive oxygen species (ROS) during metabolic processes are generated by mitochondria and result in cytotoxicity, eventual release of cytochrome c, activation of caspases and apoptosis. Thus, mitophagy is an important autophagic process to maintain the number and quality of mitochondria even in nutrient-rich conditions (Ref. 96).

CMA is different from other types of autophagy. It allows the selective removal of damaged/altered proteins under prolonged starvation or oxidative stress conditions. It does not involve phagophore formation and the target proteins directly cross the lysosomal membrane to enter its lumen. CMA selectively beholds and degrades substrate proteins containing the unique KFERQ pentapeptide sequence. Heat shock protein of 70 kDa (cytosolic chaperone protein) catches, forms the complex with the KFERQ motif present in the target protein and supplies it to the cytoplasmic tail of the lysosomal-associated membrane protein type 2A (LAMP2A) (Refs 97, 98). Upon formation of translocation complex via LAMP2A multimerization, the substrate protein gets translocated to the lysosomal matrix and crosses the membrane mediated by luminal chaperones and undergoes complete degradation (Ref. 99).

Transcriptionally upregulated autophagy-related genes (ATG) promote autophagosome formation in three major steps of macroautophagy. These steps include (i) serine kinase activity of pre-initiation complex; (ii) lipid kinase activity of initiation complex and (iii) ligase activity of ATG5/ATG12/ATG16 complex that helps in pulling of processed LC3/ATG8 to nascent phagophores for its conjugation to phosphatidylethanolamine (PE) (Ref. 100).

**Figure 4.**
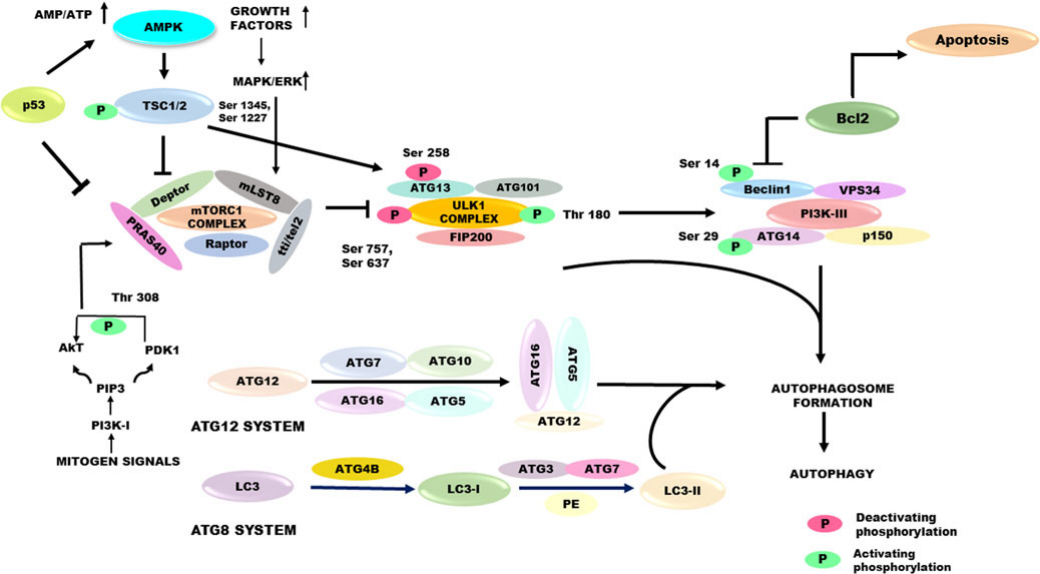
Major upstream signalling pathways that regulate autophagy – nutrient stress conditions activate AMPK or p53 signalling via TSC1/2 and inhibit mTORC1 activation. PDK1, AkT and MAPK/ERK1/2 are the upstream regulators of mTORC1 which inhibit autophagy. mTORC1 inhibition leads to an enhanced activity of the ULK1 complex and hence kinase activity of PI3K-III, which brings about autophagosome formation and hence activates autophagy. The elongation and maturation of autophagosome is facilitated by two ubiquitin-like conjugation systems – ATG8 and ATG12 which involve multiple autophagy proteins. AMPK, AMP-activated protein kinase; ATG8, autophagy-related gene 8; ATG12, autophagy-related gene 12; ERK1/2, extracellular signal regulated kinases 1/2; MAPK, mitogen-activated protein kinases; mTORC1, mTOR complex 1; PDK1, phosphoinositide-dependent kinase-1; TSC1/2, tuberous sclerosis complex 1/2; ULK1, uncoordinated-51-like protein kinase.

Autophagy initiation is mediated by four signal-sensing kinases: mammalian target of rapamycin complex 1/2 (mTORC1/2), Unc-51-like autophagy-activating kinase 1/2 (ULK1/2), AMP-activated protein kinase (AMPK) and protein kinase B (AKT or PKB) (Ref. 101). Upstream signalling pathways that regulate the autophagic process are explained in Figure 4. Pre-initiation complex is composed of ATG13, FIP200 (FAK-family interacting protein 200 kDa), ATG101 and the ULK1/ULK2 serine/threonine kinases. It is commonly considered as initiator of autophagic cascade and is negatively regulated by mTOR. The mTORC1 suppresses autophagy by phosphorylating both ULK1 and ULK2 during sufficient availability of amino acids and growth factors (Ref. 102). The pre-initiation complex is positively regulated by AMPK, an important protein kinase that detects low energy levels and activates autophagy. AMPK suppresses mTOR activity by direct phosphorylation of the raptor protein or indirect phosphorylation of the tuberous sclerosis complex 2 (TSC2) protein. AMPK can also directly activate autophagy by ULK1 phosphorylation (Ref. 103). AKT can suppress autophagy by activating its downstream target, mTORC1, during growth factor-rich condition and can stimulate autophagy by direct phosphorylation of PI3K complex proteins (Ref. 76). During nutrient-rich conditions, mTORC1 is shown to suppress autophagy by disrupting AMPK–ULK1 interaction via the phosphorylation of ULK1 (on Ser637 and Ser757) and ATG13 (Ser258). However, during nutrient-poor conditions, mTORC1 is inactivated, dephosphorylates ULK1 and separates it from mTORC1 complex. Released ULK1 is then activated by Thr180 autophosphorylation and subsequently phosphorylates other members of the ULK1 complex (Atg13, FIP200 and Atg101) (Ref. 104). Rheb (Ras homologue enriched in brain), a guanosine triphosphate (GTP)-binding protein, activates mTORC1 in a GTP-bound state. TSC1 and TSC2 interact with Rheb to inhibit its activation, causing its transition to an inactive guanosine diphosphate (GDP)-bound state. Inhibition of TSC1/2 is regulated by the PI3K/Akt or the Ras/Raf/ERK pathways in the presence of growth factors. High amino acid levels can also activate mTORC1 directly (Ref. 105). In the presence of excess amino acids, it is directed to the lysosome by the Ragulator–Rag complex. The Ragulator–Rag complex which resides on lysosome membrane works with the lysosome-linked Rheb and regulates autophagy activation (Ref. 102).

Phosphorylation of Beclin1/ATG6 by ULK1/ATG1 or ULK2 activates the lipid kinase activity of VPS34 (a class III PI3K). VPS34 (vacuolar sorting protein-34) is a catalytic component of the initiation complex (composed of ATG14L, VPS15 and other regulatory factors, in addition to Beclin1). This results in increased production of phosphatidylinositol-3-phosphate (PIP3) from PIP2. Formation of PIP3 recruits ATG5–ATG12/ATG16L-containing conjugation complex to the growing phagophore. This conjugation complex transfers processed LC3-II from ATG3 to PE and allows its integration into the lipid membranes of the growing phagophores (Refs 106, 107). Processed LC3 selects and interacts with cargo directly or indirectly via cargo-adaptor molecules containing specific motifs called LC3-interacting region motifs. One such multifunctional cargo protein is identified as sequestosome 1 (SQSTM1), also known as ubiquitin-binding protein p62, which selectively binds and delivers the ubiquitinated target contents to autophagosomes. Bcl-2, anti-apoptotic protein binds to N-terminal Bcl-2 homology 3 domain of Beclin1 to inhibit autophagy (Ref. 108). Autophagosome maturation and fusion with lysosomes is followed by degradation of autophagosomal cargo under acidic conditions. Finally, cargo constituents (nucleotides, fatty acids and amino acids) are recycled to the cytosol and are made available for various biosynthetic processes to support cell growth (Refs 106, 109).

Recent studies decipher the controversial functions of autophagy in tumourigenesis. In the early stages of carcinogenesis, autophagy can reduce the emergence of the mutagenic factors and inhibit cancer development, whereas in the middle and late stages of carcinogenesis, autophagy can resist the stress conditions and inhibit apoptosis to maintain the survival of cancer cells. The paradoxical function of autophagy as inducer of oncogenesis and suppressor of tumourigenesis is not only influenced by stage of cancer but also on environmental conditions such as state of immune system, nutrient availability, pathogenic conditions and microenvironmental stress. Basal autophagy and proper functioning of its proteins is required in tumour repression preliminary by preventing excessive ROS production which originates in damaged mitochondria (Ref. 110). Studies suggest that during later stages of tumour development, increased autophagy degrades the defective proteins and organelles, helps the tumour to overcome extreme stressful conditions such as hypoxia and nutrient deprivation, supports high metabolic demand and maintain viability of cancer cells (Refs 111, 112). Although the mechanisms underlying the pro-survival effects of tumour in later stages are largely unknown, nevertheless there are number of ongoing studies which examine the importance of autophagy in the maintenance of stemness in both normal tissue stem cells and CSCs. It is hypothesized that stress conditions in TME convert EMT tumour cells into autophagic non-cycling CSCs whereas release of paracrine factors in TME niche converts EMT tumour cells into cycling CSCs (featuring low autophagy) (Refs 113, 114). Subsequent section discusses the influence of TME on promoting autophagy and inducing stemness-like properties and metastatic potential of tumour cells.

### TME, autophagy and cancer stemness

**Figure 5.**
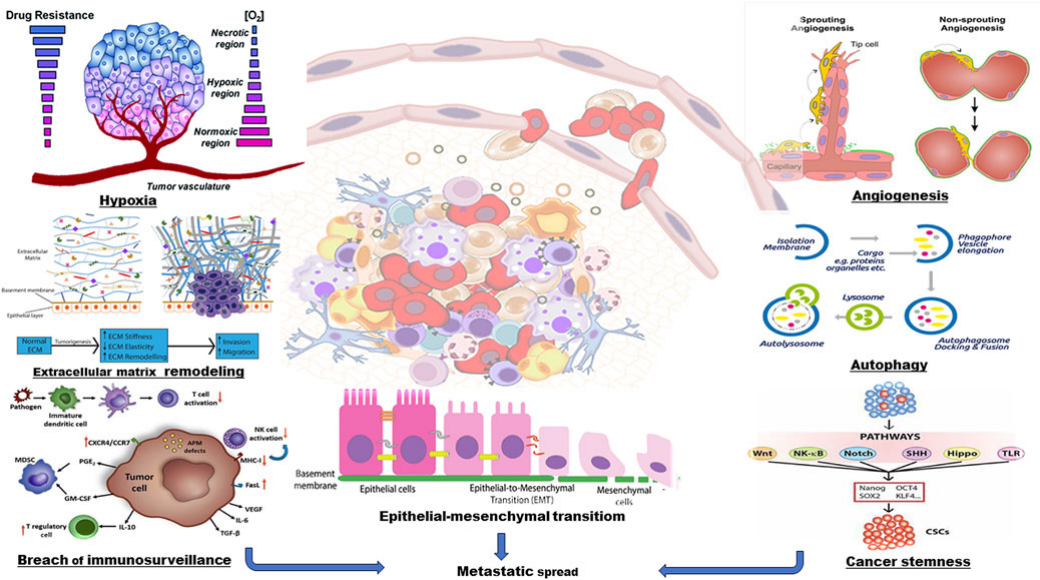
TME supports tumour development at primary and distant sites – cancer stemness, extracellular matrix remodelling, hypoxia, escape of immunosurveillance, angiogenesis and autophagy in the TME contribute to the formation of epithelial–mesenchymal transitioned cells and promote tumour development and its spread at distant sites.

Autophagy exhibits a significant degree of context dependency in cancer. It is influenced by not only the type/stage of cancer but also the local stressful microenvironment/systemic extracellular milieu of the tumour. It contributes to every stage of CSC physiology including generation, differentiation, plasticity, maintenance of stemness, breach of immune surveillance, invasion and metastasis (Fig. 5). Recent studies examine the important role of autophagy in the biology of variety of CSCs including breast, pancreatic, liver, ovarian, osteosarcoma and glioblastoma (Refs 115, 116, 117, 118, 119). Prolonged exposure to hypoxia and stressful microenvironment induces autophagy in multiple human AML cell lines and primary blasts. However, autophagy inhibition in the late stage overcomes the survival and chemoresistance of leukaemias stem cells (Ref. 120). Reprogramming of somatic cells to pluripotent stem cells with pluripotency factor SOX2, repressed mTOR expression and increased autophagy is a complex process. Maintenance of haematopoietic stem cells (HSCs) through a FOXO3A-induced autophagy survival programme and the importance of autophagy in the survival of mesenchymal stem cells and human embryonic stem cells have recently been reported (Refs 16, 121, 122). Similar to tissue stem cells, many ongoing studies strongly support the dependency of CSCs on autophagy. Higher levels of ATG4, ATG5 and Beclin1 are observed and silencing of ATG4B and ATG7 affects cell survival in CML. FIP200 depletion results in reduced phosphorylation of epidermal growth factor receptor (EGFR), decreased STAT3 activation and consequently impairs the tumourigenic potential of ALDH^+^ breast CSCs (Ref. 123). The most common CSC marker, CD133 is reported to be associated with an increased autophagic activity of CSCs (Ref. 124). CD133^+^ pancreatic cancer cells under intermittent hypoxia conditions display stem-like properties, increased autophagic flux (high Beclin1 and LC3-II), expressions of hypoxia inducible factor-1*α* (HIF-1*α*), E-cadherin, N-cadherin and vimentin and high metastatic potential (Ref. 125). An increase in autophagic flux and Beclin1 expression is noted in the ALDH^+^ CSCs derived from mammospheres compared with bulk tumour population. Characteristic properties of CD44^+^CD24^−^/low breast CSCs depend on autophagic flux. These properties include mammosphere formation, survival, invasive potential and stem-like properties. Mesenchymal phenotype of these CSCs is induced and characterized by TGF-*β*, vimentin expression, low CD24 and high CD44 (Ref. 15). TGF-*β* induces the non-cycling subpopulation of CSCs, invasion potential and increased protection against anti-cancer drugs in squamous cell carcinoma. Autophagy inhibition results in decreased TFG*β*2 and TGF*β*3 expression and defective Smad signalling and affects the CD29^hi^CD61^+^ phenotype of breast CSCs (Ref. 126). Elevated levels of autophagy and lysosomal genes and ATG4A in the mammospheres are associated with an increase in CSC number and in vivo tumourigenicity. Stemness promoting the Janus Kinase-signal transducer and activator of transcription (JAK–STAT) signalling pathway (STAT3 phosphorylation/activation) has been identified as a molecular readout of autophagy dependency in triple-negative breast cancer (Ref. 127). Another study examines the role of platelet-derived growth factor receptor (PDGFR) signalling in inducing the stemness, invasion and metastasis. PDGFR*α* inhibition is shown to reduce invasion and metastasis but not tumour growth. PDGFR is also reported to promote hypoxia-induced autophagy in non-CSCs by prolonging the half-life of HIF-1*α* (Refs 128, 129, 130). The siRNA-mediated silencing of beclin1 is significantly shown to inhibit the activation of rapamycin-induced autophagy and attenuate the invasive property of colon cancer cells (Ref. 131). Study by Qureshi-Baig *et al*. identifies the increased self-renewal capacity of CSCs or TICs derived from the patients diagnosed with colorectal cancer. Study further determines the involvement of phosphorylation of ezrin (EZR) at Thr567 residue and protein kinase C*α* (PRKCA/PKC*α*) as a potential kinase in hypoxia-induced autophagy-mediated self-renewal of CSCs (Ref. 132). Another recent study examines the higher expression of LAMP2A, a critical receptor for chaperone-mediated autophagy substrate proteins at the lysosomal membrane, in patient-derived GSCs. Its higher levels correlate with advanced glioma grade and poor overall survival and its depletion diminishes GSC-mediated tumourigenic activities (Ref. 133).

### Autophagy promotes EMT and cancer stemness

There are number of experimental studies on humans which explain the role of positive crosstalk between EMT and increased autophagic flux in conferring metastatic phenotype and poor disease outcome. Study by Jinushi *et al*. reports the effect of autophagy-mediated regulation of TGF-*β* on EMT induction in myeloid cells which increases the invasive and metastatic potential of tumour cells (Ref. 134). Further, this study reports the effect of myeloid-derived autophagy on the accumulation of M2 macrophages in tumour tissues in a colony stimulating factor-1 and TGF-*β*-dependent manner and impaired antitumour immune responses (Ref. 134). Another study by Luo *et al*. describes the role of TME in promoting autophagy which further contributes to induce EMT, produce ROS and increase the migratory and invasive potential of A549 lung adenocarcinoma cells (Ref. 135). Epithelial–mesenchymal transitioned renal carcinoma cells with CSC phenotypes exhibit resistance to chemotherapies upon autophagy activation by suppressing mTOR inhibition (Ref. 125). siRNA against ATG3 or ATG7 or inhibition of autophagy by chloroquine (CQ) results in repression of EMT in hepatocellular carcinoma (HCC). Starvation-induced autophagy is shown to increase invasion and metastasis via TGF-*β*/SMAD3 signalling in HCC (Ref. 136). ATG12 downregulation and CQ treatment is studied to increase the expression of epithelial marker CD24, decrease mesenchymal cell marker vimentin and impair the migratory and invasive potential of breast CSCs (Ref. 137). Expression of two autophagy regulators, damage-regulated autophagy modulator 1 (DRAM1) and SQSTM1 in glioblastoma CSCs correlate with the increased levels of mesenchymal factors and migratory and invasive behaviour (Ref. 138).

### Mitophagy promotes cancer stemness

Despite the presence of functional mitochondria, cancer cells rely on aerobic glycolysis, a phenomenon called ‘Warburg effect' rather than OXPHOS for energy requirement (Ref. 139). However, surrounding TME such as the hypoxic niche of solid tumours or regions with adequate levels of oxygen dictates CSCs to adapt unique metabolic programme. Study by Vlashi and Pajonk reports the dependence of glioma CSCs mainly on OXPHOS for energy supply, whereas other studies emphasize on the dependence of glioma CSCs on glycolytic intermediates for energy supply (Ref. 140). These neuronal stem cells exhibit more fragmented mitochondria and downregulation of mitochondrial respiratory activity (Ref. 141). Later studies on glioblastoma, pancreatic ductal adenocarcinoma, lung cancer side population cells, breast cancer, AML and CML suggest that CSCs rely more on OXPHOS for energy production (Refs 142, 143). Studies now report that selective degradation of damaged or superfluous mitochondria by mitophagy is highly implicated in stem cell self-renewal. It maintains HSCs in a glycolytic state, limits oxidative metabolism, makes cells less efficient in generating ATP than OXPHOS and thus contributes to the slow cycling, self-renewing phenotypic state of stem cells (Refs 144, 145). Studies report that the enhanced mitochondrial metabolism and suppressed mitophagy induces differentiation and loss of stemness (Refs 146, 147, 148). Oesophageal squamous cell carcinoma cells undergoing EMT exhibit increased mitophagy. Nevertheless, inhibiting the Parkin-dependent mitophagy causes loss of stem cell marker, CD44 and results in cell death (Ref. 15). Reduced mitochondrial mass and elimination of mitochondrial p53 are reported to be required for the maintenance of hepatic CSCs (Ref. 149). It is further examined that reduced mitophagy allows nuclear mobilization of phosphatase and tensin homologue deleted induced kinase 1 phosphorylated p53, where it antagonizes OCT4 and SOX2 induction of NANOG, the critical transcription factors of stemness (Ref. 149). HIF-1*α* is shown to transcriptionally upregulate the mitophagy receptors including BNIP3 (Bcl2 interacting protein 3), BNIP3L/NIX or FUNDC1 (Bcl2 interacting protein 3-like). These receptors interact with LC3 through their LIR motif, reduce mitochondrial mass, avoid activation of apoptosis and promote mitophagy and cancer stemness (Refs 150, 151, 152). Study by Yan *et al*. observes therapeutic resistance against doxorubicin exhibited by CD133^+^/CD44^+^ CSCs derived from HCT8 human colorectal cancer cells because of excessive rate of BNIP3L-mediated mitophagy flux (Ref. 153).

### Autophagy evades immune surveillance and promotes stemness

Studies over the past few years suggest the active participation of autophagy in the inhibition of tumour immune surveillance and allow the survival of disseminated tumour cells (DTCs) in different cancer types (Ref. 154). Inhibition of autophagy through silencing of Beclin1 is examined to restore the cytotoxic T-lymphocyte (CTL)-mediated lysis of MCF7 breast cancer cells (Ref. 154). Impaired CTL lysis in melanoma cells is attributed to autophagy via degradation of connexin 43 (Ref. 155). Reduced surface expression of major histocompatibility complex-I in pancreatic cancer cells is associated with active autophagy (Ref. 156). Another study reports the impact of autophagy inhibition in the destruction of renal cell carcinoma by NK cells (Ref. 157). Interleukin (IL)-6 secretion is important for CSC maintenance and it induces CD44^+^/CD24 low phenotype in breast cancer cell lines and tumour (Refs 50, 51). Autophagy inhibition reduces IL-6 secretion via the STAT3/JAK2 pathway. Nevertheless, IL-6/STAT3/JAK2 signal transduction is important for the conversion of non-CSCs into CSCs (Refs 50, 51). Pro-autophagic protein AMBRA1 controls stemness and regulatory T-cell differentiation and homoeostasis upstream of the FOXO3/FOXP3 axis (Ref. 158).

### Autophagy promotes tumour dormancy and metastasis

DTCs remain dormant for a longer period of time until they seed and develop new metastatic sites. DTCs are reported to be in autophagic state where autophagy promotes their survival during dormancy. Autophagy allows DTCs to switch from dormant state to growth state (Ref. 159). Its inhibition depletes the dormant cells but leaves the population of proliferating tumour cells intact (Ref. 159). Effect of down expressed aplasia Ras homologue member I (ARHI) tumour suppressor on dormancy and reduced autophagy in ovarian tumours is observed. Further observations on re-expression of ARHI in ARHI-deficient SKOv3 ovarian cancer cells induced autophagy and blocked tumour growth conclude autophagy-dependent enforced expression of ARHI in dormant cells. Autophagy inhibition may cause elimination of DTCs and avert metastasis (Ref. 160).

DTCs in the bone marrow of breast cancer patients express CSC markers and sustain autophagy-dependent survival. These features of dormant tumour cells greatly resemble to quiescent and motile CSCs (Refs 160, 161). Dormant tumour cells of mouse model of pancreatic ductal adenocarcinoma survive K-Ras inactivation, promote tumour re-growth, display increase in autophagy and exhibit CSCs features including high CD44 expression, potential to form tumour spheroids and increased tumour initiation properties in vivo (Ref. 162).

Autophagy supplies key metabolites, turns over key transcription factors, ensures reversible quiescent state and prevents irreversible senescence and thus promotes dormant stem-like state. Activation of liver kinase B1–AMP-activated protein kinase (LKB1–AMPK) signalling via a p27^Kip1^-dependent growth arrest in G1 of the cell cycle; activation of the AMPK-induced pre-initiation complex and AMPK-dependent ULK1 phosphorylation promote autophagy and maintain the cells in quiescent and viable states (Refs 163, 164). Loss of p27^Kip1^ resulting in rapid apoptotic cell death under metabolic stress and LKB1–AMPK signalling is examined as a mechanism of autophagy induction, growth arrest and cell survival (Ref. 163). Given the role of CSCs in metastasis, therapy resistance and disease recurrence, mechanisms/strategies leading to autophagy inhibition and suppression of dormant/stemness phenotype of tumour cells may offer better treatment options to combat metastasis.

## Blocking autophagy and cancer stemness: therapeutic prospects

High tumourigenic potential of CSCs poses a huge challenge in the development of targeted cancer therapies. The presence of subpopulation of CSCs results in the continual evolution of tumour cells. Advancements in single-cell technologies lead to the discovery of increasing number of biomarkers specific to CSCs and important biochemical pathways. Nevertheless, existing targeted therapies are not sufficient for complete eradication of tumours because of their high regeneration potential. Autophagy is studied as one of the key pathways that supports the maintenance of subpopulations of CSCs by increasing the availability of recycled nutrients. Recent ongoing studies characterize autophagic functions in CSC aggressiveness and therapeutic resistance. Number of theories and mechanisms behind the therapeutic induction of autophagy are explained.

### Mechanisms of therapeutic resistance

Conventional or non-conventional treatments targeting PI3K, AKT or mTOR activity result in autophagy derepression (Ref. 165). This derepression/activation could be regulated by both mTOR complexes (mTORC1 and mTORC2). The study further demonstrates that blocking autophagy and the use of inhibitors of PI3K/AKT/mTOR signalling axis promote cell death in early or late stages (Ref. 165). Another study explains DNA damage-induced p53-mediated induction of autophagy regulators such as DNA DRAM1 following the treatment with conventional genotoxic agents, such as radiation or cisplatin (Ref. 166). Mitochondrial damage because of increased production of ROS and an endoplasmic reticulum stress response because of protein aggregation following cancer therapy result in autophagy induction and higher levels of ATG5, LC3 and other autophagic genes (Refs 12, 167, 168). Another study by Ojha *et al*. describes the significance of JAK-mediated autophagy in preserving the stemness in cisplatin-resistant bladder cancer cells (Ref. 169). Another study reports selective increase in autophagic flux in drug-resistant bladder cancer cells. Its pharmacological or siRNA-mediated inhibition specifically potentiates the chemotherapeutic effects of gemcitabine, mitomycin and cisplatin-resistant bladder CSCs (Ref. 170). Autophagy suppresses apoptosis and contributes to chemotherapy, radiotherapy and immune resistance in CSCs. Inhibition of lysosome-mediated autophagy increases the sensitivity of nasopharyngeal carcinoma stem cells to radiation therapy (Ref. 171). ATG5 activation in the absence of glutamine prevents radiation-induced damage. Prostate CSCs with high glutamine are shown to possess radioresistant properties (Ref. 172). Specific mechanisms of drug chemoresistance in CSCs upon activation of autophagy are not yet fully explored. Nevertheless, studies report the impact of blocked autophagy in reduced chemoresistance via GRP78/*β*-linked protein/ABCG2 axis in breast CSCs. The SOX2-*β*-catenin/Beclin1/autophagy signalling axis, GSK-3*β*/Wnt/*β*-catenin-linked protein signalling and PIK3C3/VPS34 activation are shown to promote chemoresistance in colorectal CSCs. mTOR inhibition promotes apoptosis in glioma stem cells and HCC whereas BRCA1 regulates apoptosis, cell cycle progression and autophagy thereby affect drug sensitivity in ovarian CSCs (Ref. 173). Evasion of immune surveillance by CSCs supports their survival. Control of miR-155 and activation of TRAIL and autophagy inhibition support increased CD4 cancer infiltrating lymphocyte expression. Autophagy-mediated degradation of MHC-I promotes immune escape in pancreatic cancer cells. Stimulation of the NANOG–LC3B–EGFR axis promotes autophagy and immune resistance. Another study examines the effect of interaction of ATG7 and IL-6 receptors with the macrophages (TAM) on androgen deprivation therapy resistance in prostate CSCs (Ref. 173).

Recent studies establish the relationship among the increase in autophagy and cancer stemness in response to cancer therapies. Reducing the plasticity of CSCs rather than hitting them directly by blocking autophagy could be the powerful therapeutic strategy to kill tumour cells (Ref. 174). Besides, metabolic symbiosis is shown to exist among CSCs, non-CSCs and CAFs residing in TME. It is hypothesized that targeting non-CSCs and CAFs with autophagic inhibitors may result in reduced availability of nutrients to CSCs and thus negatively impacts the survival of CSCs.

### Autophagy inhibition in cancer treatment

Autophagy inhibition is shown to reduce clonogenic survival of breast, lung and cervical cancer cell lines following irradiation treatment (Ref. 174). Higher mammalian sterile 20-like kinase 4 (MST4) activity induced phosphorylation and ATG4B protease activation is examined to be associated with increased autophagic flux in human GSCs. This results in increased self-renewal properties, sphere formation and increased in vivo tumourigenesis. CQ blocks autophagy by getting trapped in the lysosome, promoting alkalinization of the lysosomes and inhibiting lysosomal acid protease activity. Blocking autophagy with CQ and reducing ATG4B activity promote the therapeutic effects of radiation in a glioblastoma (GBM) transplant model (Ref. 175). Inhibition of autophagy with CQ results in accumulation of higher levels of FoxO3a in tumour cells and increased expression of pro-apoptotic target gene Puma. This promotes the synergistic killing of tumour cells with genotoxic agents, including doxorubicin and etoposide, in combination with CQ (Ref. 176). Combination of 5-fluorouracil, CQ and Notch inhibitor reduces cell viability and enhances therapeutic sensitivity of gastric CSCs (Ref. 177). Knockdown of ATG7 is shown to potentiate the inhibitory effect of salinomycin on survival of glioma and AML CSCs (Ref. 178). Study by Qureshi-Baig *et al*. determines the hypoxia–autophagy–PKC–EZR signalling axis as a novel regulatory mechanism in reducing the colorectal CSCs. Genetic targeting of autophagy or pharmacological inhibition of PRKC/PKC and EZR results in a decreased tumour-initiating potential of TICs and CRC progression (Ref. 132). An autophagy inhibitor, Autophinib acts by inhibiting lipid kinase VPS34, is examined as a potential agent in A549 human lung CSCs. Remarkable downexpression of core stem cell factors, Sox2 and Oct4 on Autophinib-treated A549 cancer cells correlates with pronounced induction of apoptosis and inability to form spheroids (Ref. 179). Owing to a strong link between autophagy signalling and cellular plasticity, studies are focusing on therapeutic interventions by blocking autophagy in CSCs expressing increased mesenchymal markers. Autophagy inhibition by downregulation of ATG12 and CQ treatment is examined to increase the expression of CD24 (epithelial marker) and decrease vimentin (mesenchymal cell marker), impaired migratory and invasion potential of breast CSCs (Ref. 15).

### Translational implications

Number of ongoing clinical trials examines the positive outcome of disease therapy by manipulating autophagy in complementation with conventional therapeutic agents and it represents the promising target for counteracting CSCs aggressiveness. Nevertheless, CSC heterogeneity, tumour and patient specificity further complicate the choice of novel drug combinations in order to completely eradicate the CSC population or inhibit their proliferation. Nowadays, more potent inhibitors of autophagy other than CQ are being explored to develop combination of different therapies. These include E64d (an inhibitor of cathepsins B, H, and L, D and E) or pepstatin A and concanamycin A (a selective inhibitor of V-ATPase that prevents lysosome and endosome acidification) (Ref. 180). As these are the lysosome inhibitors and hence, cannot affect the autophagosome formation and cargo sequestration. Thus, the biggest drawback with these inhibitors is that they cannot reduce the rate of mitochondrial sequestration by autophagosomes, thereby preserve the CSC effects that rely on mitophagy and limit the therapeutic efficacy of combinational drugs on tumour cell killing. Thus, the drugs that can inhibit the initial phases of autophagy such as VPS34 or ULK1 inhibitors could provide better results. The most studied cancer types in FDA-approved CQ trials include brain, breast, lung and gastrointestinal tract. Blocking autophagy within the tumour is shown to have a moderate effect on tumour progression. Nevertheless, autophagy inhibition via oral administration of CQ results in more substantial reduction in tumour growth and invasion (Ref. 181). Phase I/II demonstrates migration of immune cells (macrophages) into the ducts and significant reduction in tumour in ductal carcinoma in situ (DCIS). Studies on phase II randomized controlled trials speculate that CQ supplementation in a combination therapy with antineoplastic agents is more beneficial in reducing proliferation in breast cancer treatment (Ref. 182). Autophagy modulation with safe and tolerable high-dose hydroxychloroquine and dose-intense temozolomide is associated with a reduced tumour growth in the treatment of patients with advanced solid tumours and melanoma (Ref. 183). Recently multiple studies on clinical trials demonstrate the synergistic effect of autophagy inhibitors in reducing proliferation and antineoplastic agents/chemotherapeutic/immunotherapies agents in creating a cytotoxic environment to disrupt the cancer homoeostasis (Ref. 184). Table 2 summarizes various clinical trials investigating autophagy inhibitor drugs in combination with chemotherapies, radiotherapies and/or immunotherapies in various cancer types (Refs 185, 186, 187, 188, 189, 190, 191, 192, 193, 194, 195).

**Table 2.** Clinical trials investigating the drugs inhibiting autophagy in combination with chemo/radiation/immunotherapies in various cancer types

Intervention	Cancer type	Phase	Adverse effects	References
CQ + TMZ + RT	Brain	I (13 patients)	Irreversible blurred vision, nausea, electrocardiogram QT-corrected interval prolonged	185
CQ + bortezomib + cyclophosphamide	Multiple myeloma	II (11 patients)	Fatigue, constipation, myalgia, nausea, cough, neuropathy, diarrhoea, dyspnoea, congestion, anorexia, thrombocytopenia, neutropenia	186
CQ + radiation	Non-small cell lung cancer	II (73 patients)	None	187
CQ + TMZ + RT	Glioblastoma	II (30 patients)	None	187
CQ + taxane	Breast	II (38 patients)	Adverse effects reported in patients with grade ⩾ 3	188
CQ + carmustine	Glioblastoma	III (18 patients)	None	189
HCQ + VOR	Colorectal cancer	I (19 patients)	Gastrointestinal disturbances, fatigue	190
HCQ + VOR	Refractory multiple myeloma	I (25 patients)	Cytopenia, gastrointestinal toxicity	191
HCQ + TMZ	Melanoma	I (40 patients)	Diarrhoea, anorexia, constipation, fatigue, nausea	183
HCQ + gemcitabine	Pancreatic	I/II (20 patients)	Lymphopenia, elevated alanine aminotransferase	192
HCQ + carmustine + RT + CQ	Glioma	II (30 patients)	None	187
HCQ + paclitaxel + carboplatin + bevacizumab	Non-small cell lung cancer	II (40 patients)	None	193
HCQ + gemcitabine + Nab-paclitaxel	Pancreatic cancer	II (34 patients)	None	194
HCQ + everolimus	Renal cell carcinoma	II (33 patients)	None	195
HCQ + everolimus	Breast	II (60 patients)	None	187

CQ, chloroquine; HCQ, hydroxychloroquine; RT, radiation therapy; TMZ, temozolomide; VOR, vorinostat.

Further investigations on tumour type, stage and grade-specific quantification of autophagic flux in patients' samples and their dependency on CSCs survival would help in predicting the state of autophagic activation. This would systematically guide to explore and develop the effective novel inhibitors for autophagy-targeting therapies. As the autophagy inhibitor drugs (mono/combinational therapies) utilized in clinical setting are recently being tested in cancer treatment and therefore, their potential long-term side effects are not fully evaluated. Although autophagy inhibition acts as tumour suppressor in malignant therapeutics but it might be protective in other diseases such as cardiomyopathy, liver diseases, autoimmune disorders and neurodegenerative diseases. Besides this most of the trials do not indicate the side effects if any. Hence exploring the multiple novel drug combinations with minimal side effects if any in clinical setting is deemed necessary.

## Funding statement

This work was supported by Indian Council of Medical Research (ICMR), Govt. of India (grant no. 5/3/8/24/2020-ITR).
